# On the relationship between maxillary molar root shape and jaw kinematics in *Australopithecus africanus* and *Paranthropus robustus*

**DOI:** 10.1098/rsos.180825

**Published:** 2018-08-29

**Authors:** Kornelius Kupczik, Viviana Toro-Ibacache, Gabriele A. Macho

**Affiliations:** 1 Max Planck Weizmann Center for Integrative Archaeology and Anthropology, Deutscher Platz 6, Leipzig 04103, Germany; 2 Department of Human Evolution, Max Planck Institute for Evolutionary Anthropology, Deutscher Platz 6, Leipzig 04103, Germany; 3 Facultad de Odontología, Universidad de Chile, Sergio Livingstone Pohlhammer 943, Independencia, Región Metropolitana, Santiago de Chile, Chile; 4 School of Archaeology, University of Oxford, Oxford OX1 3QY, UK; 5 Department of Earth and Planetary Sciences, Birkbeck, University of London, London WC1E 7HX, UK

**Keywords:** Plio-Pleistocene hominins, *Australopithecus africanus*, *Paranthropus robustus*, *Paranthropus boisei*, tooth root splay, dietary ecology

## Abstract

Plio-Pleistocene hominins from South Africa remain poorly understood. Here, we focus on how *Australopithecus africanus* and *Paranthropus robustus* exploited and—in part—partitioned their environment. Specifically, we explore the extent to which first maxillary molar roots (M^1^) are oriented and thus, by proxy, estimate the direction of loads habitually exerted on the chewing surface. Landmark-based shape analysis of M^1^ root reconstructions of 26 South African hominins and three East African *Paranthropus boisei* suggest that *A. africanus* may have been able to dissipate the widest range of laterally directed loads. *Paranthropus robustus* and *P. boisei*, despite having overlapping morphologies, differ in aspects of root shape/size, dento-cranial morphologies, microwear textures and C4 food consumption. Hence, while *Paranthropus* monophyly cannot be excluded, equivalence of dietary niche can. The South African hominins occupied distinct ecological niches, whereby *P. robustus* appears uniquely adapted to dissipate antero-posteriorly directed loads.

## 1. Background

Nearly a century ago, *Australopithecus africanus* was first announced from South Africa [1]. Despite extensive fossil discoveries since then, South African hominins have remained enigmatic. It is unclear whether the morphological variability within the *A. africanus* hypodigm indicates the presence of multiple species [2] or should be expected in a long-lived, eurybiomic taxon [3]. Nor do we know the phylogenetic relationship(s) of South and East African hominins [4,5]. To understand the evolutionary pathway(s) of the South African Plio-Pleistocene hominins and their role in human evolution, it is imperative to resolve issues concerning their diversity and specializations, particularly with regard to feeding ecology. Here, we focus on *A. africanus* and *Paranthropus robustus* who were perhaps—although only for a short period of their evolutionary history—synchronic [6–9] and apparently subsisted on similar proportions of C4 foods [10–12]. Specifically, we investigate an understudied functional–morphological aspect of the masticatory apparatus, namely tooth root splay and its bearing on mandibular kinematics.

Diet plays a crucial role in all aspects of a species’ biology, whereby differences in dietary ecology—however subtle—are essential when primates are sympatric within the same physical habitat [13]. One of the ways to accomplish niche partitioning (or differentiation) is through selection for larger body size in one of the species, as seen in *Gorilla* and *Pan*, for example [14]. Recent evidence suggests that *A. africanus* may have been slightly larger than *P. robustus* [15] but, compared to the great apes, body size differences between these South African hominins are subtle and other mechanisms must therefore have been realized to achieve niche partitioning. Indeed, despite the overlap in carbon isotope composition of the dental tissues that suggests consumption of similar proportions of C3/C4 foods [10–12], there are marked differences in craniodental morphology [16,17], tooth wear [17,18], microwear textures [19,20] and aspects of enamel microstructures [21] between *A. africanus* and *P. robustus* that attest to different feeding strategies. Interpreting these morphological differences is difficult however. First, *A. africanus* and *P. robustus* overlapped for only a short period of time, and different habitat specificities through time might be expected. Second, despite its assumed shared ancestry with East African *P. boisei*, *P. robustus* is not only unique in cranial morphology but, surprisingly, has no (known) modern or extinct analogue as regards enamel microstructure and microwear textures (but see [22]); this makes it difficult to ascertain *P. robustus*' dietary ecology. Third, and perhaps most problematic, *A. africanus* is a poorly defined, although apparently long-lived taxon, which may in fact contain multiple species. Taken together therefore, the phylogeny and the extent to which the South African hominins exploited their habitat during the Plio-Pleistocene are far from resolved.

**Figure 1.**
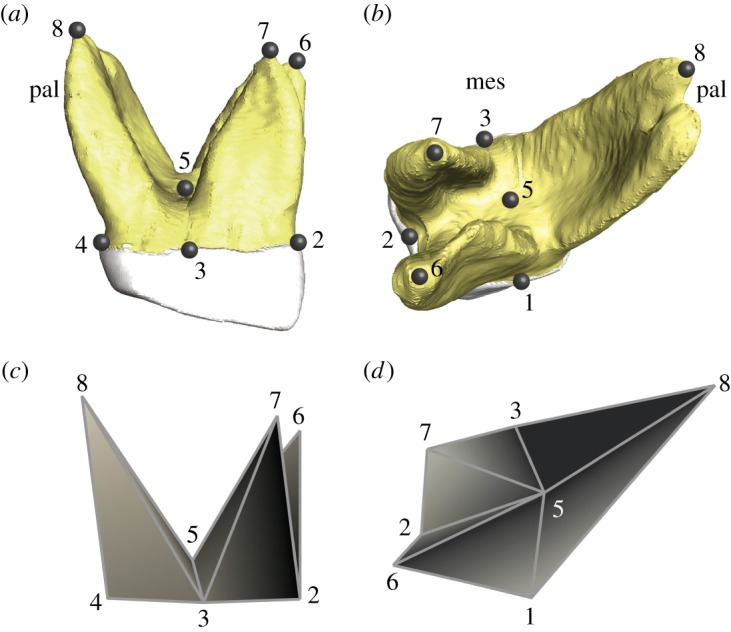
Maxillary first molar root with landmark configuration. (*a*,*b*) Three-dimensional reconstructed maxillary first molar with eight landmarks. (*c*,*d*) Polygon representation of root shape. (*a*,*c*) Lateral view. (*b*,*d*) Latero-superior view. Landmarks: 1, midpoint of distal cervix (enamel–cementum junction); 2, midpoint of buccal cervix; 3, midpoint of mesial cervix; 4, midpoint of palatal cervix; 5, centre of root furcation; 6, tip of distobuccal root; 7, tip of mesiobuccal root; 8, tip of palatal root. mes, mesial; pal, palatal.

Tooth root orientation and splay promises to provide novel insights into South African hominin dietary ecology. Masticatory forces generated at the occlusal surface must be dissipated into the jaw through the tooth roots. This is most efficiently achieved by aligning the force vector parallel with the long axis of the tooth, thus preventing potentially damaging bending and shear forces along the roots [23,24]. With this in mind, the characteristic change in modern human tooth root orientation from anterior to posterior is unsurprising: it mirrors the changes in force vectors resulting from orofacial geometry and muscle orientation [25,26]. Furthermore, the three-rooted maxillary molars of primates are subjected to a greater range of medio-laterally directed loads than the two-rooted mandibular molars, which tend to act as pestles during the power stroke and are therefore sturdier overall [25,27]. Owing to the greater lateral excursion of the mandible anteriorly, i.e. farther away from the jaw joint, first maxillary molars must resist a wider range of differently directed loads than posterior molars [28,29]; indeed, there is a gradual decrease in maxillary molar root spread from first to third molars [25]. Hence, despite a phylogenetic component to tooth root morphology and number [30–32], variations in tooth root size and shape will reflect differences in bite force magnitude and orientation [25,33–37], thus informing adaptations towards specific feeding regimens. To accomplish the latter, it needs to be borne in mind, however, that the lateral excursion of the mandible is not only constrained by orofacial geometry, but will adjust in accord with the rheology of foods consumed, whereby plastic foods will lead to an increased lateral amplitude of the mandible [38–40]. Conceivably, habitual consumption of such foods will select for greater bucco-palatal divergence of maxillary first molar tooth roots. Here, we extend these observations to relate root splay and overall morphology of first maxillary molars (M^1^) to jaw kinematics in South African *A. africanus* and *P. robustus* using landmark-based geometric morphometrics (figure 1). We enquire (i) whether the partially sympatric South African hominins exploited a similar dietary niche, (ii) whether the niche partitioning in South African hominin taxa is comparable to that of sympatric extant West African great apes, *Gorilla gorilla* and *Pan troglodytes*, or (iii) whether *P. robustus* and *P. boisei* from East Africa are likely to have shared an ecological niche.

## 2. Material and methods

### 2.1. Sample

The M^1^s of a sample of 21 West African extant great apes (*G. gorilla* (five males, five females); *Pan troglodytes* (six males, five females)) as well as of 26 fossil hominins from South Africa (14 *A. africanus* from Makapansgat and Sterkfontein and 12 *P. robustus* from Kromdraai and Swartkrans)^1^ and, for comparison, of three *P. boisei* from Kenya (West Turkana and Chesowanja) and Tanzania (Olduvai) to indirectly address issues of phylogenetic constraints in *P. robustus* were segmented from computed tomography scans (CTs) in Avizo 9.01 (FEI, Hillsborough, OR, USA) (electronic supplementary material, table S1 and figures S1 and S2; figure 1). High-resolution CTs were not available for other relevant East African hominin fossils, thus restricting the overall sample size available. The South African and Kenyan fossil craniodental material studied here are derived from collections at the Ditsong National Museum of Natural History in Pretoria and the University of Witwatersrand, Johannesburg (both in South Africa) and the National Museums of Kenya, Nairobi, and was scanned with the BIR ACTIS 225/300 μCT-scanner of the Max Planck Institute for Evolutionary Anthropology in Leipzig [48]. For OH5, a previously published segmentation of the M^1^ tooth roots derived from medical CT scans was used [49]. CT images of great ape skulls housed at the Muséum National d'Histoire Naturelle (Paris) were scanned at AST-RX (Accès Scientifique à la Tomographie à Rayons X). The isometric voxel sizes of the great ape and fossil CT images (except for OH5) ranged from 0.10 to 0.16 and 0.03 to 0.09 mm, respectively. The CT dataset of OH5 had a pixel size of 0.48 mm and a slice thickness of 1 mm [49]. Fossil specimens were selected based on the state of preservation of the roots and visibility of the root outlines in the CT scans.

### 2.2. Landmarking

A total of eight landmarks representing main features of root shape (splay, tilt and relative size of root trunk and root portions) were collected in Avizo for each molar around the neck of the tooth (cervix) as well as on the furcation of the roots and the tips of the three roots (two buccal and one palatal; figure 1). A detailed anatomical description of the landmarks is provided in the caption of figure 1. The positioning of landmarks was verified by two observers (K.K. and V.T.-I.).

### 2.3. Morphometric analyses

**Figure 2.**
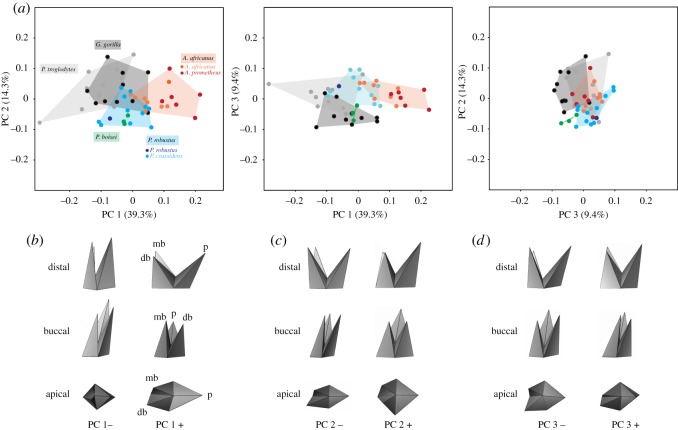
Maxillary first molar root shape variation in 29 South and East African fossil hominins and 21 West African great apes. (*a*) Shape space revealed in PC analysis. (*b*–*d*) Polygons showing root shape extremes along the three PCs in three anatomical views. db, distobuccal root; mb, mesiobuccal root; p, palatal root.

Centroid sizes of the fossils and great apes by species were compared using the Mann–Whitney *U* pairwise comparisons with and without the Bonferroni correction. Landmark data were transformed into shape variables (Procrustes coordinates) by generalized least squares Procrustes superimposition. Principal components analysis (PCA) of Procrustes coordinates was used to generate a morphospace of M^1^ root shape variation. All analyses were done in PAST v. 3.14 [50]. In order to visualize the shape variation represented by each principal component (PC), a simplified PLY surface was created (figure 2) and warped to the extreme of the PCs using the ‘geomorph’ package [51] in R [52]. The mean shapes for the five taxonomic groups (*A. africanus, P. robustus, P. boisei, Pan troglodytes* and *G. gorilla*) were compared using canonical variates analysis (CVA) and the statistical significance was assessed with a permutation test (10 000 rounds) in MorphoJ [53]. Allometry, the dependence of shape variation on changes in size, was appraised via multivariate regression of shape variables on centroid size [54] and its statistical significance was determined (10 000 permutations) in MorphoJ. As a discriminant analysis in MorphoJ (10 000 permutations) did not reveal any significant differences in root shape between male and female gorillas (Procrustes distance = 0.07; *p* = 0.66) and chimpanzees (Procrustes distance = 0.12; *p* = 0.10), respectively, we did not take into account sexual dimorphism in any of the subsequent analyses.

## 3. Results

The first PC explains 39% of the M^1^ root shape variance (figure 2*a*; electronic supplementary material, tables S2 and S3) and reflects variation in bucco-palatal root splay (figure 2*b*, distal view), mesio-distal tilting of the roots (figure 2*b*, buccal view) and mesio-distal expansion of the root base (figure 2*b*, apical view). PC 2 (14% of the variance; figure 2*a*; electronic supplementary material, table S3) accounts for differences in the relative proportion of the palatal and the buccal roots (figure 2*c*, distal view) and the orientation of the distobuccal root (figure 2*c*, apical view), while PC3 (9% variance; figure 2*a*; electronic supplementary material, table S3) reflects differences in the height of the interradicular furcation (i.e. the point where the tooth splits into the three roots portions; figure 2*d*, distal view) and the mesio-distal spread of the buccal roots (figure 2*d*, apical view).

When the taxonomic groups are considered, we find that *A. africanus* and *P. robustus* are more distinct from one another than are the sympatric great apes, i.e. there is less overlap (figure 2*a*). *Australopithecus africanus* exhibits roots that are splayed bucco-palatally and aligned vertically in buccal view (figure 2*a,b*; electronic supplementary material, figure S1). Although fossils within the *A. africanus* cluster have been attributed to a second species, *A. prometheus*, the overall variation observed for *A. africanus* does not exceed that seen in extant great apes. Hence, we did not explore the possibility of a second species further in the present study. The majority of the *P. robustus* and the three *P. boisei* fossils have both negative PC1 and PC2 scores which are associated with equally proportioned buccal and palatal roots with a moderate bucco-palatal root splay. The overall distally inclined roots, when viewed buccally, distinguishes them from *A. africanus* (figure 2*b*,*c*). When PC3 is considered, *P. boisei* forms a separate cluster outside the *P. robustus* shape space and this is associated with a lower furcation and more diverging buccal roots (figure 2*a*,*d*). As regards the great apes, *Pan troglodytes* shows a low degree of bucco-lingual divergence and both the palatal and buccal roots are tilted distally, while *G. gorilla* has a moderate bucco-palatal root splay and mesio-distally diverging buccal roots (figure 2*a*,*b*; electronic supplementary material, figures S1 and S2). A CVA based on Procrustes distances confirms the observed grouping pattern in the PCA, i.e. mean shapes are significantly different within and between the two South African fossil hominins and the great apes; there is no statistical difference for *P. boisei*, probably due to the group's small sample size (table 1).

**Table 1.** Differences among group mean shapes based on Procrustes distances (lower diagonal) and statistical significance after 10 000 rounds of permutations (upper diagonal). Bold values indicate statistical significance after the Bonferroni correction.

	*Australopithecus africanus*	*Paranthropus robustus*	*Paranthropus boisei*	*Pan troglodytes*	*Gorilla gorilla*
*Australopithecus africanus*		**<0**.**001**	**<0**.**001**	**<0**.**001**	**<0**.**001**
*Paranthropus robustus*	0.12		0.06	**<0**.**001**	**<0**.**001**
*Paranthropus boisei*	0.16	0.10		0.01	0.01
*Pan troglodytes*	0.21	0.12	0.16		**0**.**001**
*Gorilla gorilla*	0.16	0.13	0.13	0.12	

**Figure 3.**
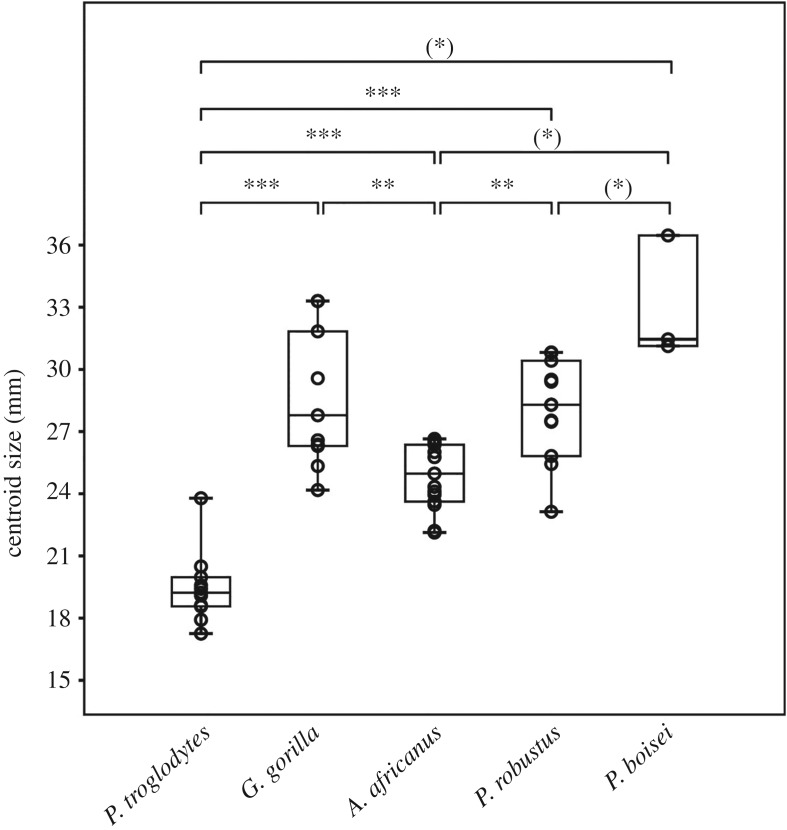
Centroid sizes of great ape and Plio-Pleistocene fossil hominins. The Bonferroni-corrected significant differences between group medians are indicated by asterisks (***p < 0.001, **p < 0.01, *p < 0.05; asterisks in brackets indicate uncorrected significant *p*-values). The Mann–Whitney *U* pairwise test results are listed in electronic supplementary material, table S4.

Although there are significant M^1^ root size differences among the fossil hominins (*P. boisei* > *P. robustus* > *A. africanus*) and between the great apes (*G. gorilla* > *Pan troglodytes*) (figure 3; electronic supplementary material, table S4), centroid size accounts for only 7% of shape variation (*p* = 0.006) as revealed by a multivariate regression. Large M^1^ roots as in *P. boisei* and some *G. gorilla* correspond to more evenly sized buccal and palatal root portions, while small roots—as seen in *Pan troglodytes*—have a narrower root splay and a relatively large palatal root portion (electronic supplementary material, figure S3).

## 4. Discussion

This study aimed to explore whether differences in craniodental morphologies between the Plio-Pleistocene hominins from South Africa *A. africanus* and *P. robustus* could initially have been driven by niche partitioning or may—perhaps—be due to phylogenetic constraints in the latter taxon. To do so, we focused on tooth root form (shape and size) and—in particular—tooth root splay, a functionally important trait that may inform whether a species habitually consumes foods that need to be broken down in crush (axial loading) or in shear (lateral loading). Analyses reveal clear differences between all extant and extinct primates, indicative of distinct dietary adaptations, whereby *A. africanus* has the most bucco-palatally splayed M^1^ roots compared to the other species (figure 2). What is more, the distinction between the M^1^ root shapes of the South African hominin taxa is more pronounced than is that between the sympatric great apes, even though the hominins, unlike chimpanzees and gorillas, overlap in root centroid size (figure 3) and body mass [15,55].

African great apes are essentially frugivorous [56] and are mostly confined to tropical forests, whereas hominins have evolved into more open habitats [57] and have adopted a more eclectic feeding strategy [58]. The relatively small (compared to *G. gorilla*) and slender, supero-inferiorly oriented tooth roots of *Pan troglodytes* (figures 2 and 3) are consistent with vertical low-force biting when masticating soft fruits. While sympatric *Pan troglodytes* and *G. gorilla* largely overlap in fruit consumption, the latter are more versatile in their diet, which includes tougher foods such as fibrous fruits and foliage [56,59]. Fibrous (tough) foods require a greater lateral stroke of the mandible to be broken down than do soft fruits [39,60]. Indeed, the sturdier M^1^ roots with a coronally positioned furcation of *G. gorilla* seem to reflect the consumption of more demanding foods compared to *Pan troglodytes* [33,61], and/or perhaps more repetitive chewing of these tougher foods [62]. The greater molar root surface areas of *G. gorilla* similarly seems to indicate higher estimated bite forces in this species when compared with *Pan troglodytes* [33,61,63] or repetitive chewing [37].

Extrapolating from great ape ecology and their relative positions within the morphospace (figure 2), the larger M^1^ root divergence in conjunction with asymmetrically worn crowns observed in *A. africanus* (electronic supplementary material, figure S4) suggests that this species engaged in an increased lateral stroke of the mandible to consume more ductile (tougher) foods than *P. robustus*, albeit—presumably—at a lower bite force, as judged by the smaller roots (see [63]). By contrast, the overall larger M^1^ roots with similar-proportioned buccal and palatal roots in *P. robustus* (and *P. boisei*) seem to imply more vertical (axial) loading at, perhaps, higher bite forces [61,63,64]. When viewed within the broader context of the orofacial skeleton, these results provide more nuanced insights into the dietary adaptations of early Pleistocene hominins.

The relationship between tooth crown size and root size reflects dietary preferences among anthropoid primates [37,65], such that hard object feeders that employ high bite forces have relatively large molar root surface areas for a given occlusal area than do soft object feeders. As M^1^ crown base areas in *A. africanus* and *P. robustus* are similar [66], whereas the roots are larger in the latter taxon, it is plausible that *P. robustus* teeth were subjected to higher occlusal loads *in vivo*. Such an interpretation is in accord with a higher occurrence of three-rooted maxillary fourth premolars in *P. robustus* when compared with *A. africanus* [32]; this would make these teeth well suited to dissipate comparatively higher loads. In line with such a proposition, *P. robustus,* like *A. africanus* but unlike *P. boisei*, displays a strengthening of the anterior face, i.e. an anterior pillar, which is probably an adaptation to higher bite forces, too [16,67]. High vertically directed loads in *P. robustus* are also implicated by the apically oriented enamel prisms in relation to phase I and phase II wear facets [21], which will render the dental tissue stiff. The latter observation, together with the distinct, i.e. relatively flat, postcanine mesowear pattern clearly distinguishes *P. robustus* from *A. africanus* [17,68] (electronic supplementary material, figure S4) and suggests predominantly uni-axial loading in the former. Given that *P. boisei* exhibits relatively flatly worn molars, too [69] (electronic supplementary material, figure S4), it is tempting to infer similarity in dietary ecology between *P. robustus* and *P. boisei.* Such an interpretation seems highly unlikely, however, not least because of the different ecological settings in which *P. robustus* and *P. boisei* lived [70,71].

Although both *P. robustus* and *P. boisei* exhibit flat molar wear patterns, their teeth differ in morphology and size [69], as well as in significant aspects of tooth root morphology (figure 2*a*,*c*,*d*). Jaw kinematics and the mechanism by which these two taxa achieved their flat molar wear most certainly differed. In contrast with *P. robustus, P. boisei* apparently engaged in horizontally directed repetitive chewing [63,72,73], as can be inferred from the species’ derived temporo-mandibular joint (TMJ) with its prominent articular eminence also [74]. Such a joint morphology facilitates a more equal separation of the upper and lower tooth row (i.e. the TMJ would not have acted as a simple hinge during jaw opening), as it does in modern humans, but, unlike modern humans, also makes possible an increased horizontal medio-lateral movement of the mandible [72,74]; such an arrangement is advantageous when grinding starch-rich, abrasive foods. The hyper-thick enamelled teeth of *P. boisei* are most probably an adaptation to high rates of dental wear [72]. However, *P. boisei* lacks substantial amounts of enamel prism decussation, thus making the dental tissue vulnerable to tensile forces; this argues against substantial amounts of laterally directed loads that are necessary when masticating tough foods [72]. Conversely, there are relatively high levels of enamel prism decussation in *P. robustus* teeth [75], suggestive of multi-directional loading. This is at odds with their flat molar wear though, which implies predominantly uni-axial loading. Closer inspection of the species’ tooth roots makes it possible to reconcile what—at first—appears to be a contradiction: unlike *P. boisei*, *P. robustus* displays overall distally facing M^1^ roots and converging buccal roots (figure 2*a*,*c*). This unusual tooth root morphology explains the apparent contradiction and raises questions about the foods consumed by *P. robustus*.

*Paranthropus robustus* has a unique microwear texture pattern among primates [10,58,76] which is particularly distinct from that observed in *P. boisei* [77]*.* Overall, *P. robustus*' microwear texture seems perhaps closest to *Papio cynocephalus* [75,76], but this papionin does not exhibit the specialized cranial morphologies of *P. robustus*. Yet, as regards the carbon isotope composition of dental tissue, *P. robustus* is not distinct from either *A. africanus* or the papionins but from *P. boisei* [10]. This makes it challenging to infer the food sources consumed by *P. robustus.* As it is unlikely that *P. robustus* occupied a dietary niche to the exclusion of all other primates (both extant and extinct), the possibility must therefore be entertained that *P. robustus* simply comminuted food in a different manner, e.g. through an increased antero-posterior component to the mandibular stroke. Such a suggestion is reasonable on the grounds of *P. robustus’* mandibular morphology [17] combined with a lack of the derived *P. boisei* TMJ morphology in this taxon [74]. To what extent the relatively marked expression of the curve of Spee in *P. robustus* (e.g. DNH 7, see [78]) compared with both *A. africanus* and *P. boisei* may have facilitated or mitigated an antero-posterior and/or rotational movement of the mandible cannot be resolved here and will be subject of further investigation. Regardless, propositions of a more three-dimensional movement of the mandible in *P. robustus* are not far-fetched given (i) that all masticatory strokes have a slight antero-posterior component and (ii) that the TMJ, which links the mandible with the cranium, has six degrees of freedom, thus allowing for mandibular movement (rotation and translation) in all directions. Such a morphological set-up also implies that the masticatory apparatus is a redundant system, whereby the same outcomes, i.e. comminution of foods, can be achieved through different mechanisms, i.e. mandibular strokes. This compounds dietary reconstructions and highlights that the answer to the question about *P. robustus* diet may not so much lie in *what* they were eating, but in *how*. Only a multi-disciplinary approach has the potential to throw light on the ‘what’, although some general conclusions are nonetheless possible.

All primates including anthropoids are behaviourally flexible in their food choices [56], and may have been particularly so in the more open, fluctuating environments occupied by early hominins [79]. Furthermore, primate species select foods in accordance with the capabilities of their masticatory apparatus [80] and are capable of adjusting the oral processing of these foods in such a way as to extract nutrients otherwise inaccessible [81,82]. It is the combined effects of these mechanisms that will define the dietary niche(s) of primates. Elucidating this complexity requires a multi-disciplinary approach, whereby determining the manner in which early hominins chewed (kinematics) is likely to hold important clues. This is highlighted by the results obtained for *P. robustus*. Whatever the habitual (as yet unidentified) foods of this species may turn out to have been, it is clear from its tooth root morphology that this hominin occupied a dietary niche distinct from that of *P. boisei* and probably from *A. africanus*, too*.* Importantly, however, the foods habitually consumed not only required a different mechanism for breakdown, but were most likely of relatively high quality and abundant. The latter is suggested on the basis that *P. robustus* had absolutely and relatively larger brains than either *A. africanus* or *P. boisei* [15,83]. Building and maintaining large brains would have necessitated the consumption of higher quality (and abundant) foods. Ultimately, the combined effects of food selection and larger brains (behavioural flexibility) seem to have afforded *P. robustus* the opportunity to adjust to fluctuating environmental conditions and to survive well after the other taxa had become extinct, i.e. into the mid-Pleistocene Climate Transition.

## Supplementary Material

Table S1

## Supplementary Material

Table S2

## Supplementary Material

Table S3

## Supplementary Material

Table S4

## Supplementary Material

Fig. S1

## Supplementary Material

Fig. S2

## Supplementary Material

Fig. S3

## Supplementary Material

Fig. S4

## Footnotes

1 We are aware that some researchers argue for the presence of two species within the *A. africanus* hypodigm, i.e. they make a distinction between *A. africanus* and *A. prometheus* [2,41–43]. Also, the *P. robustus* hypodigm has originally been divided into *P. robustus* from Kromdraai and *P. crassidens* from Swartkrans [44–47]; most researchers now accept that these fossils belong to the single taxon *P. robustus*.

## Data accessibility

All morphometric data (both centroid size and 3D landmark coordinates per specimen) are provided in the electronic supplementary tables S2 and S3. The supplementary figures illustrate the 3D tooth models from which the 3D landmark coordinates were taken. The original CT scans of the fossils and the great apes from which the tooth models were generated are property of the respective museums in South and East Africa as well as France and cannot be made publicly available by the authors of this contribution.
