# Supplementary material for: On the relationship between maxillary molar root shape and jaw kinematics in Australopithecus africanus and Paranthropus robustus
Source: R Soc Open Sci. 2018 Aug 29;5(8):180825. doi: 10.1098/rsos.180825 (PMC6124107; doi:10.1098/rsos.180825)
Supplement: Table S1 [file rsos180825supp1.docx]

Table S1. Fossil sample

| Taxon | Accession no | Site | Deposit | Side |
| --- | --- | --- | --- | --- |
| *A. africanus* | MLD 6 | Makapansgat | Makapansgat | R |
|  | MLD 9 | Makapansgat | Makapansgat | R |
|  | Sts 8 | Sterkfontein | Member 4 | L |
|  | Sts12 | Sterkfontein | Member 4 | L |
|  | Sts 52a | Sterkfontein | Member 4 | L |
|  | Sts 53 | Sterkfontein | Member 4 | R |
|  | Sts 71 | Sterkfontein | Member 4 | R |
|  | Stw 13 | Sterkfontein | Member 4 | L |
|  | Stw 183 | Sterkfontein | Member 4 | L |
|  | Stw 252j | Sterkfontein | Member 4 | L |
|  | Stw 280 (283) | Sterkfontein | Member 4 | L |
|  | Stw 498a | Sterkfontein | Member 4 | R |
|  | TM 1511 | Sterkfontein | Member 4 | R |
|  | TM 1512 | Sterkfontein | Member 4 | R |
| *P. robustus* | SK 13.14 | Swartkrans | Member 1 Hanging Remnant | L |
|  | SK 17 | Swartkrans | Member 1 Hanging Remnant | R |
|  | SK 46 | Swartkrans | Member 1 Hanging Remnant | L |
|  | SK 47 | Swartkrans | Member 1 Hanging Remnant | R |
|  | SK 48 | Swartkrans | Member 1 Hanging Remnant | L |
|  | SK 49 | Swartkrans | Member 1 Hanging Remnant | R |
|  | SK 52 | Swartkrans | Member 1 Hanging Remnant | R |
|  | SK 57 | Swartkrans | Member 1 Hanging Remnant | L |
|  | SK 83 | Swartkrans | Member 1 Hanging Remnant | L |
|  | SKW 8 | Swartkrans | Member 1 Hanging Remnant | R |
|  | SKW 11 | Swartkrans | Member 1 Hanging Remnant | R |
|  | TM1517a | Kromdraai | Kromdraai B | L |
| *P. boisei* | KNM-WT 17400 | West Turkana |  | R |
|  | KNM-CH 1 | Chesowanja |  | R |
|  | OH 5 | Olduvai |  | L |
