# Supplementary material for: On the relationship between maxillary molar root shape and jaw kinematics in Australopithecus africanus and Paranthropus robustus
Source: R Soc Open Sci. 2018 Aug 29;5(8):180825. doi: 10.1098/rsos.180825 (PMC6124107; doi:10.1098/rsos.180825)
Supplement: Table S2 [file rsos180825supp2.docx]

Table S2. Centroid size (in mm) and scores of the first three PCs of fossil hominin and great ape M^1^ root shape

| **Taxon** | **Accession no** | **Centroid size** | **PC 1** | **PC 2** | **PC 3** |
| --- | --- | --- | --- | --- | --- |
| *A. africanus* | TM1511 | 24.33 | 0.122 | -0.024 | -0.024 |
|  | TM1512 | 23.95 | 0.103 | -0.025 | 0.007 |
|  | Sts12 | 22.21 | 0.019 | 0.005 | 0.053 |
|  | Sts52a | 24.97 | 0.050 | 0.002 | 0.045 |
|  | Sts71 | 23.47 | 0.135 | 0.006 | 0.004 |
|  | Stw183 | 26.36 | 0.105 | -0.007 | 0.016 |
|  | Stw498a | 26.01 | 0.226 | 0.014 | -0.036 |
|  | Sts8 | 23.63 | 0.058 | -0.011 | 0.060 |
|  | Sts53 | 25.44 | 0.052 | -0.041 | 0.001 |
|  | Stw13 | 25.76 | 0.005 | 0.014 | 0.052 |
|  | Stw252 | 24.11 | 0.129 | 0.100 | 0.021 |
|  | Stw283 | 26.51 | 0.150 | -0.019 | -0.022 |
|  | MLD6 | 22.13 | 0.127 | 0.056 | 0.035 |
|  | MLD9 | 26.65 | 0.210 | -0.062 | 0.030 |
| *P. robustus* | SK13.14 | 30.79 | 0.071 | -0.093 | -0.023 |
|  | SK48 | 28.30 | 0.004 | -0.080 | 0.033 |
|  | SKW11 | 27.53 | 0.042 | -0.047 | 0.012 |
|  | SK17 | 25.43 | -0.025 | 0.037 | 0.096 |
|  | SK46 | 30.42 | -0.089 | -0.085 | 0.024 |
|  | SK47 | 23.13 | -0.015 | 0.008 | 0.070 |
|  | SK49 | 28.29 | 0.064 | -0.032 | 0.043 |
|  | SK52 | 30.82 | -0.005 | -0.037 | -0.019 |
|  | SK57 | 27.47 | 0.004 | 0.025 | 0.096 |
|  | SK83 | 29.41 | 0.052 | -0.025 | -0.017 |
|  | TM1517a | 25.82 | -0.061 | -0.064 | 0.042 |
|  | SKW8 | 29.50 | -0.096 | -0.075 | 0.009 |
| *P. boisei* | KNM-WT 17400 | 31.13 | -0.015 | -0.082 | -0.071 |
|  | KNM-CH1 | 31.45 | -0.017 | -0.074 | -0.046 |
|  | OH5 | 36.46 | -0.004 | -0.054 | -0.022 |
| *P. troglodytes* | 1940_1192, male | 23.79 | -0.285 | -0.078 | 0.049 |
|  | 1947_0051, male | 20.49 | -0.150 | -0.034 | 0.023 |
|  | 1952_0144, male | 19.41 | -0.129 | 0.089 | -0.025 |
|  | 1962_1477, male | 19.09 | -0.048 | -0.026 | -0.030 |
|  | 2007_1449, male | 17.25 | -0.023 | 0.068 | -0.054 |
|  | 0000_A116, male | 19.23 | -0.094 | 0.016 | -0.009 |
|  | 1940_0467, female | 19.58 | -0.096 | 0.009 | 0.033 |
|  | 1947_0149, female | 19.97 | -0.135 | 0.035 | 0.006 |
|  | 1947_0224, female | 18.61 | -0.083 | 0.045 | 0.030 |
|  | 1962_1481, female | 18.57 | -0.039 | 0.097 | -0.049 |
|  | 1982_0002, female | 17.92 | 0.014 | 0.146 | 0.080 |
| *G. gorilla* | 1906_0441, male | 33.30 | 0.060 | -0.045 | -0.062 |
|  | 1929_0503, male | 31.84 | -0.106 | -0.014 | 0.019 |
|  | 1939_0438, male | 26.37 | -0.020 | 0.047 | -0.084 |
|  | 1949_0524, male | 33.30 | -0.077 | -0.008 | -0.057 |
|  | 1987_0250, male | 25.34 | -0.069 | 0.138 | 0.007 |
|  | 1933_0075, female | 26.58 | -0.021 | 0.086 | -0.048 |
|  | 1936_1985, female | 27.78 | -0.133 | 0.026 | -0.093 |
|  | 1962_1496, female | 26.30 | 0.011 | -0.007 | -0.062 |
|  | 1963_0276, female | 24.18 | 0.060 | 0.088 | -0.074 |
|  | 1940_0373, female | 29.57 | -0.039 | -0.009 | -0.068 |
