# Supplementary material for: On the relationship between maxillary molar root shape and jaw kinematics in Australopithecus africanus and Paranthropus robustus
Source: R Soc Open Sci. 2018 Aug 29;5(8):180825. doi: 10.1098/rsos.180825 (PMC6124107; doi:10.1098/rsos.180825)
Supplement: Table S3 [file rsos180825supp3.docx]

Table S3. Mann-Whitney *U* pairwise test results for centroid size comparison. Uncorrected p-values are shown; bold values indicate statistical significance after Bonferroni correction.

|  | *P. troglodytes* | *G. gorilla* | *A. africanus* | *P. robustus* | *P. boisei* |
| --- | --- | --- | --- | --- | --- |
| *P. troglodytes* |  | **<0.001** | **<0.001** | **<0.001** | <0.05 |
| *G. gorilla* | 0 |  | **<0.01** | 0.974 | 0.151 |
| *A. africanus* | 4 | 20 |  | **<0.01** | <0.05 |
| *P. robustus* | 1 | 59 | 22 |  | <0.05 |
| *P. boisei* | 0 | 6 | 0 | 0 |  |
