# Supplementary figures and images for: On the relationship between maxillary molar root shape and jaw kinematics in Australopithecus africanus and Paranthropus robustus
Source: R Soc Open Sci. 2018 Aug 29;5(8):180825. doi: 10.1098/rsos.180825 (PMC6124107; doi:10.1098/rsos.180825)

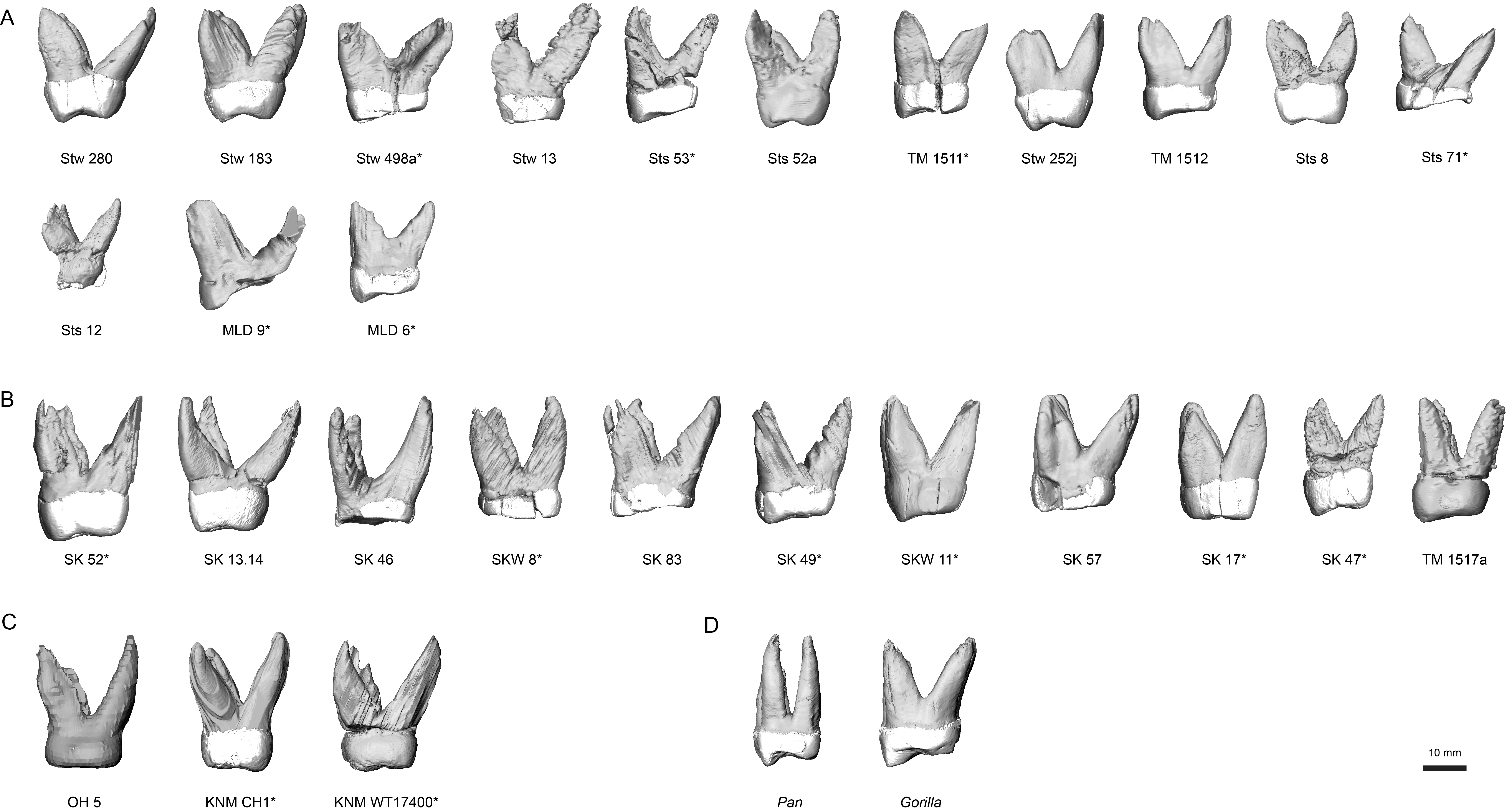

Supplement: Fig. S1 [file rsos180825supp5.jpg]

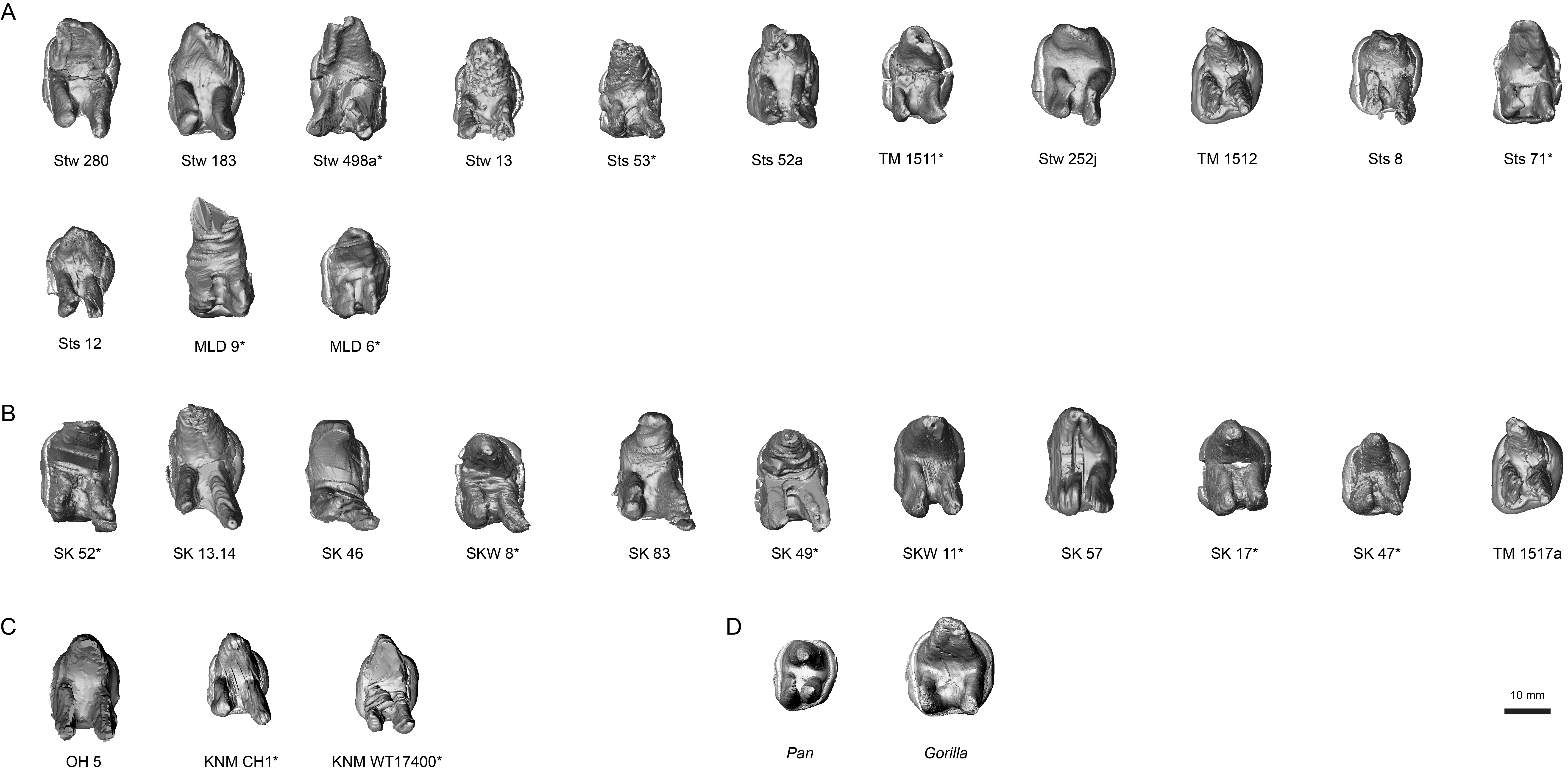

Supplement: Fig. S2 [file rsos180825supp6.jpg]

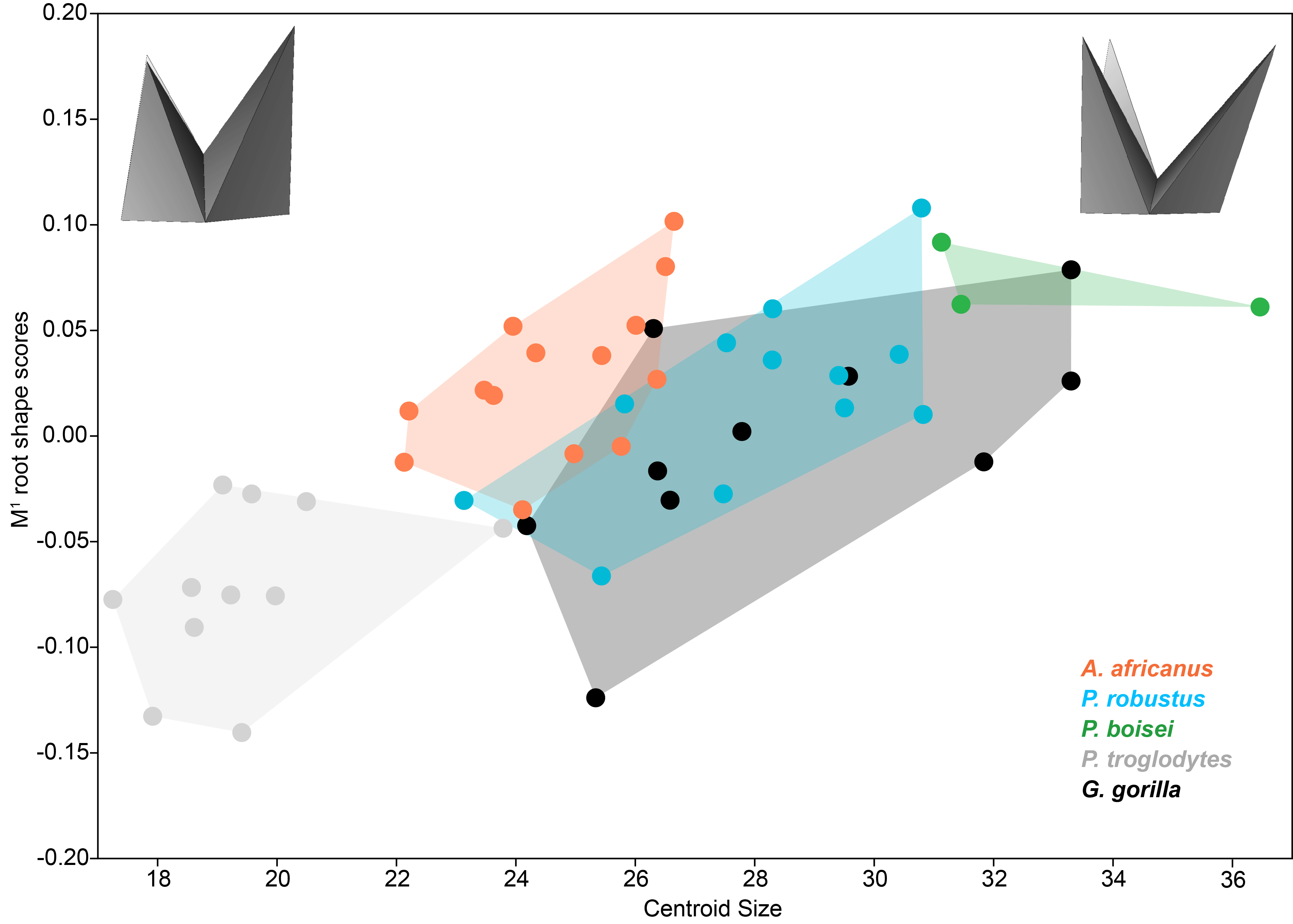

Supplement: Fig. S3 [file rsos180825supp7.jpg]

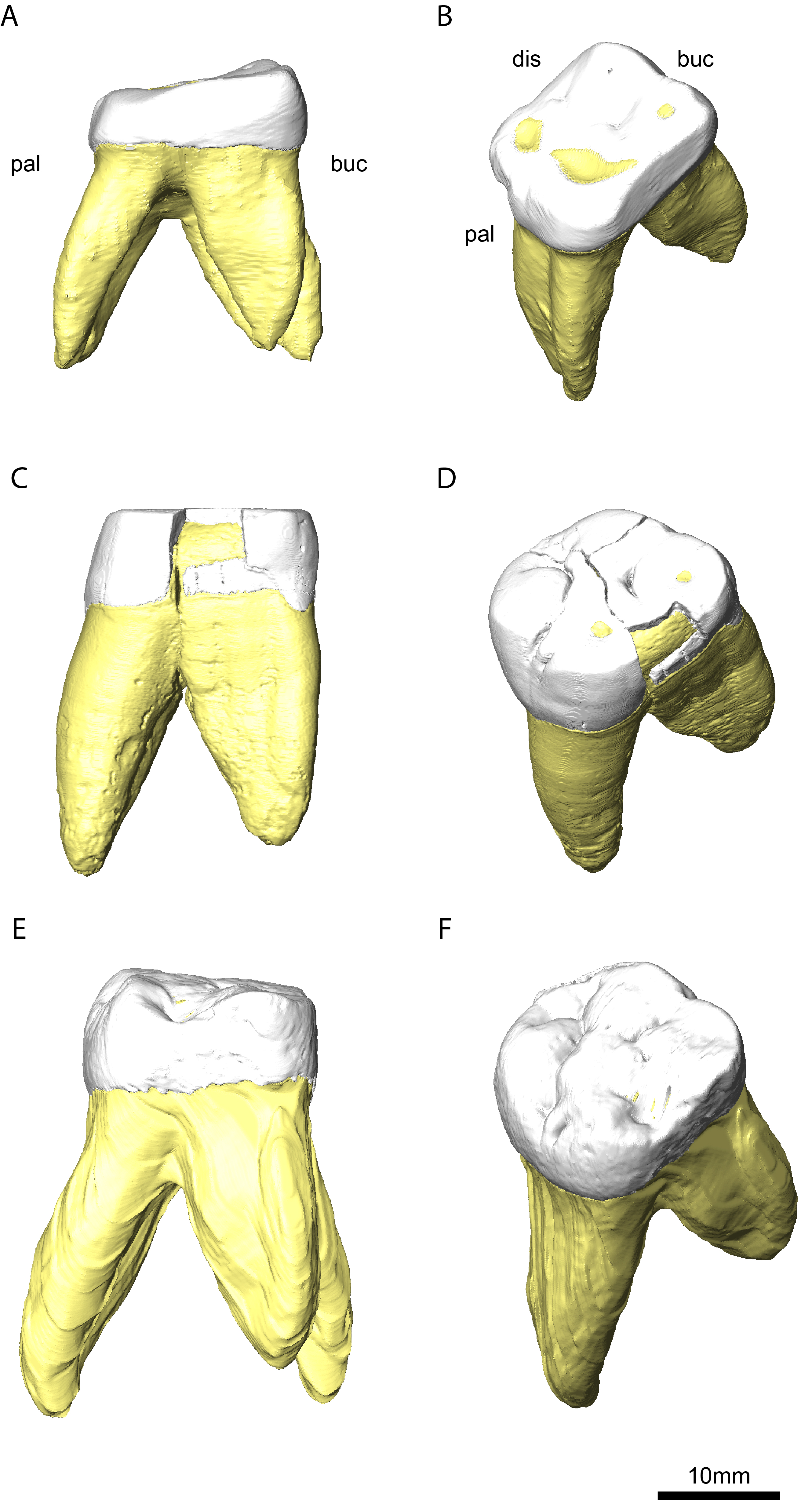

Supplement: Fig. S4 [file rsos180825supp8.jpg]
